# The Role of Chest Compressions on Ventilation during Advanced Cardiopulmonary Resuscitation

**DOI:** 10.3390/jcm12216918

**Published:** 2023-11-03

**Authors:** Izaskun Azcarate, Jose Antonio Urigüen, Mikel Leturiondo, Camilo Leonardo Sandoval, Koldo Redondo, José Julio Gutiérrez, James Knox Russell, Pia Wallmüller, Fritz Sterz, Mohamud Ramzan Daya, Sofía Ruiz de Gauna

**Affiliations:** 1 Group of Signal and Communications, Bilbao School of Engineering, University of the Basque Country UPV/EHU, Plaza Torres Quevedo 1, 48013 Bilbao, Spain; joseantonio.uriguen@ehu.eus (J.A.U.); mikel.leturiondo@ehu.eus (M.L.); koldo.redondo@ehu.eus (K.R.); josejulio.gutierrez@ehu.eus (J.J.G.); sofia.ruizdegauna@ehu.eus (S.R.d.G.); 2 Department of Applied Mathematics, Bilbao School of Engineering, University of the Basque Country UPV/EHU, Plaza Torres Quevedo 1, 48013 Bilbao, Spain; 3 Unidades Tecnologicas de Santander, Bucaramanga, Santander 680005, Colombia; csandoval@correo.uts.edu.co; 4 Center for Policy and Research in Emergency Medicine (CPR-EM), Department of Emergency Medicine, Oregon Health & Science University, Portland, OR 97239, USA; james.k.russell@mac.com (J.K.R.); dayam@ohsu.edu (M.R.D.); 5 Department of Emergency Medicine, Medical University of Vienna, 1090 Vienna, Austria; pia.wallmueller@gmail.com (P.W.); fritz.sterz@meduniwien.ac.at (F.S.)

**Keywords:** cardiopulmonary resuscitation (CPR), ventilation, ventilation rate, tidal volume, airway flow, chest compressions, advanced life support (ALS)

## Abstract

**Background:** There is growing interest in the quality of manual ventilation during cardiopulmonary resuscitation (CPR), but accurate assessment of ventilation parameters remains a challenge. Waveform capnography is currently the reference for monitoring ventilation rate in intubated patients, but fails to provide information on tidal volumes and inspiration–expiration timing. Moreover, the capnogram is often distorted when chest compressions (CCs) are performed during ventilation compromising its reliability during CPR. Our main purpose was to characterize manual ventilation during CPR and to assess how CCs may impact on ventilation quality. **Methods:** Retrospective analysis were performed of CPR recordings fromtwo databases of adult patients in cardiac arrest including capnogram, compression depth, and airway flow, pressure and volume signals. Using automated signal processing techniques followed by manual revision, individual ventilations were identified and ventilation parameters were measured. Oscillations on the capnogram plateau during CCs were characterized, and its correlation with compression depth and airway volume was assessed. Finally, we identified events of reversed airflow caused by CCs and their effect on volume and capnogram waveform. **Results:** Ventilation rates were higher than the recommended 10 breaths/min in 66.7% of the cases. Variability in ventilation rates correlated with the variability in tidal volumes and other ventilatory parameters. Oscillations caused by CCs on capnograms were of high amplitude (median above 74%) and were associated with low pseudo-volumes (median 26 mL). Correlation between the amplitude of those oscillations with either the CCs depth or the generated passive volumes was low, with correlation coefficients of −0.24 and 0.40, respectively. During inspiration and expiration, reversed airflow events caused opposed movement of gases in 80% of ventilations. **Conclusions:** Our study confirmed lack of adherence between measured ventilation rates and the guideline recommendations, and a substantial dispersion in manual ventilation parameters during CPR. Oscillations on the capnogram plateau caused by CCs did not correlate with compression depth or associated small tidal volumes. CCs caused reversed flow during inspiration, expiration and in the interval between ventilations, sufficient to generate volume changes and causing oscillations on capnogram. Further research is warranted to assess the impact of these findings on ventilation quality during CPR.

## 1. Introduction

Cardiopulmonary resuscitation (CPR) focuses on maintaining circulation and oxygenation of patients in cardiac arrest, pursuing restoration of spontaneous circulation (ROSC) [1,2]. Careful ventilation during CPR is key for adequate oxygen administration and carbon dioxide elimination [3]. Insufficient ventilation can result in hypoxemia, hypercapnia or acidosis [4,5,6], while hyperventilation is associated with adverse haemodynamic effects during cardiac arrest [7,8,9]. Also, high tidal volumes or inspiratory flows can produce high airway pressures, increasing the risk of gastric inflation and pulmonary aspiration [3,10].

Current 2020 resuscitation guidelines support the use of mouth-to-mouth or bag-mask ventilation and of advanced airway, using either a supraglottic airway or endotracheal intubation [11,12]. For non-intubated patients, guidelines recommend alternating cycles of 30 chest compressions (CCs) with two rescue breaths. After intubation, guidelines recommend ventilation at a rate of 10 breaths/min with uninterrupted CCs. Each breath should take about 1 s of inspiration time and be enough to produce a visible chest rise (approximately 500–600 mL) [12,13]. It is also recommended to closely monitor the quality of ventilation once ROSC has been achieved [14] and to monitor oxygenation to avoid episodes of hypoxia or hyperoxia, which are associated with poor outcomes [15,16].

There is increasing interest in advanced monitoring of the quality of manual ventilation during CPR [3,17]. However, the data available in the clinical setting are normally very limited. In fact, the ventilation rate is the only parameter routinely monitored by the advanced life support (ALS), generally using capnography [18,19,20]. Although waveform capnography is currently the reference for ventilation rate monitoring in intubated patients, it has severe limitations. To begin with, the capnogram fails to provide detailed information regarding other ventilation components such as tidal volumes, inspiration and expiration times, airway flow or airway pressure [17]. Recording these ventilation parameters requires specific measurement equipment [21,22], and clinical studies compiling these data are scarce [3,23]. Moreover, CCs often induce oscillations on the capnogram that are synchronised with the frequency of CCs [24]. This phenomenon limits the accuracy of the measured ventilation rate and end-tidal CO_2_ (ETCO_2_) value [24,25]. In addition, pressure changes in the thorax caused by CCs produce passive ventilations with tidal volumes insufficient for adequate gas exchange [22,26,27,28]. However, there is little information on the relationship between the effects of CCs on capnography, airway flow and volume signals.

In this context, the purpose of this observational retrospective study was twofold. First, we sought to characterize manual ventilation during ALS in intubated adult patients suffering from cardiac arrest. To that end, we calculated several parameters from signals extracted from two different databases. Second, we focused on studying the effect of continuous CCs on ventilation. Specifically, we analysed whether the amplitudes of the oscillations induced in the capnogram were related to the depth of the CCs and to the pseudo-volumes generated by the CCs. We also examined how airflow and volume are altered by CCs during the ventilatory cycle, and how this impacts the capnogram. Our research may contribute to increased knowledge on optimal management of ventilation.

## 2. Materials and Methods

### 2.1. Data Collection

Episodes included in the study were collected from two databases. Several episodes were extracted from a large database of adult out-of-hospital cardiac arrest (OHCA) recordings collected from 2011 through 2016 by Tualatin Valley Fire & Rescue (TVF&R), an ALS first response emergency medical services (EMS) agency serving eleven incorporated cities in Oregon, USA. This database is part of the Resuscitation Outcomes Consortium (ROC) Epidemiological Cardiac Arrest Registry. The second database consists of episodes of cardiac arrest collected by the Department of Emergency Medicine, Medical University of Vienna (MUV), from 2012 through 2014 [29]. Most patients were transported to the intensive care unit (ICU) by the ambulance service after suffering OHCA. Patients experiencing cardiac arrest within the hospital outside ICU were also transported internally to the Department of Emergency Medicine’s ICU. Patients were included in the study if they were in cardiac arrest on arrival to the ICU and underwent CPR. Data collections were approved by the Oregon Health & Science University (OHSU) Institutional Review Board (IRB00001736, approved on 10 October 2005) and by the Ethik kommission of Medizinische Universität Wien (EK-Nr: 1574/2012, approved on 3 September 2012, [https://ekmeduniwien.at/core/catalog/2012, (accessed on 1 November 2023)]), respectively. These databases are hereinafter referred to as TVF&R and MUV, respectively.

Episodes from the TVF&R database were recorded with Heartstart MRx monitor-defibrillators (Philips Healthcare, Andover, MA, USA), equipped with real-time CPR feedback technology (Q-CPR, Philips Healthcare, USA). CCs and ventilations were provided manually. Ventilation was provided with a bag-valve-mask or an advanced airway, either endotracheal tube or the King LT-D (supraglottic) device. Capnography was acquired using sidestream technology (Microstream, Oridion Systems Ltd., Jerusalem, Israel).

For the MUV database, a NICO Cardiopulmonary Management System monitor (Novametrix Medical Systems Inc., Wallingford, CT, USA) was used to record capnography, ventilation flow, ventilation volume and airway pressure, and finger photoplethysmography (PPG). In addition, arterial blood pressure (ABP), measured from a catheter in the radial artery, was recorded using a Philips Intellivue^®^ (Philips GmbH, Vienna, Austria) bedside monitor. CCs were applied either manually or mechanically using LUCAS^®^ devices. Endotracheal intubation was used for airway management. After intubation, ventilations were provided manually until the patient was connected to a mechanical ventilator.

### 2.2. Data Inclusion

#### 2.2.1. TVF&R Database

The signals of interest required for the study were the capnogram, the compression depth signal measured by the Q-CPR technology (based on force and acceleration sensors), and the transthoracic impedance (TI) signal acquired from defibrillation pads. Episodes with at least 20 min of continuous and concurrent signals, and with a minimum of 500 CCs, were included in the study [24]. All signals were visually inspected using custom-made software tools (Matlab R2021a) (Matlab^®^, Natick, MA, USA). Intervals with unreliable raw TI signal or capnogram caused by disconnections or excessive noise were discarded.

#### 2.2.2. MUV Database

Recordings with continuous and concurrent airflow, volume, capnogram, ABP and PPG signals were extracted from the MUV database, and visually inspected using custom-made Matlab software tools (Matlab R2021a). Only patients whose recordings showed intervals with manual ventilations during ongoing CCs were included in the study. Intervals with disconnections or excessive noise were discarded. The transition from manual to automated ventilation was assessed by locating the instant where the airflow waveform changed to the characteristic pattern of automated ventilation. The presence of CCs was confirmed by observing the ABP and PPG signals.

### 2.3. Data Annotation and Methodology

In this section, we describe the specific procedure of signal processing and data annotation defined for each of the analyses conducted in this study.

#### 2.3.1. Assessment of Ventilation Quality Parameters

**Figure 1:**
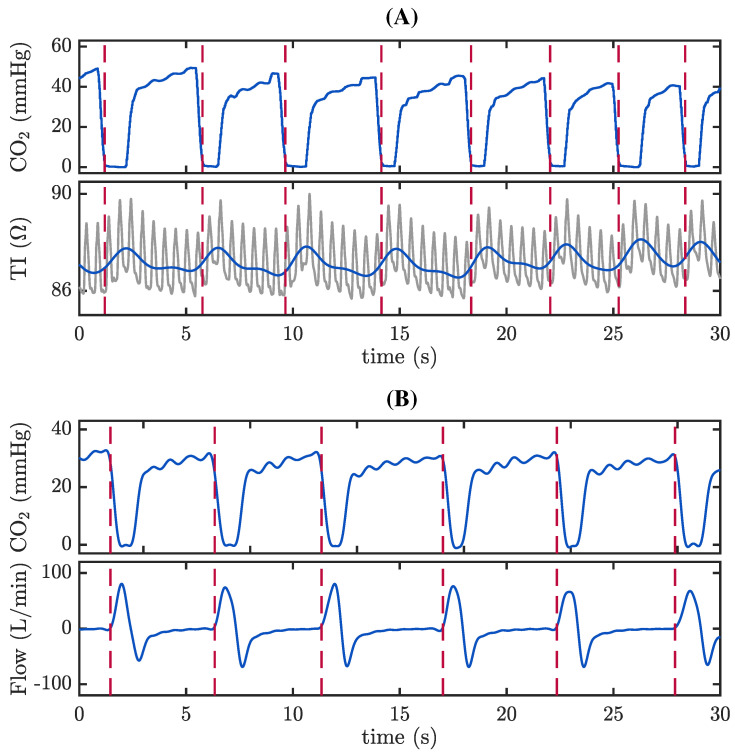
Example of ventilation annotation in the TVF&R (**A**) and MUV (**B**) databases. The red dashed lines represent each individual ventilation. (**A**) Ventilations were annotated using the capnogram (top) and the TI signal (bottom). The raw TI (grey) was low pass filtered to suppress fast oscillations caused by CCs and enhance the ventilation TI component (blue). (**B**) Ventilations annotated using the capnogram (top) and the airflow signal (bottom).

Individual ventilations were manually identified using the capnogram and the TI signal in the TVF&R database (Figure 1, panel A). The TI was low pass filtered to suppress the fast oscillations caused by CCs and thus emphasize the characteristic fluctuation associated with each ventilation (blue tracing in the bottom graph in panel A). Similarly, we annotated ventilations using the capnogram and the airflow signal in the MUV database (Figure 1, panel B). Ventilation rate was measured as the inverse of the time interval between consecutive ventilations, expressed in ventilations per minute (vpm).

**Figure 2:**
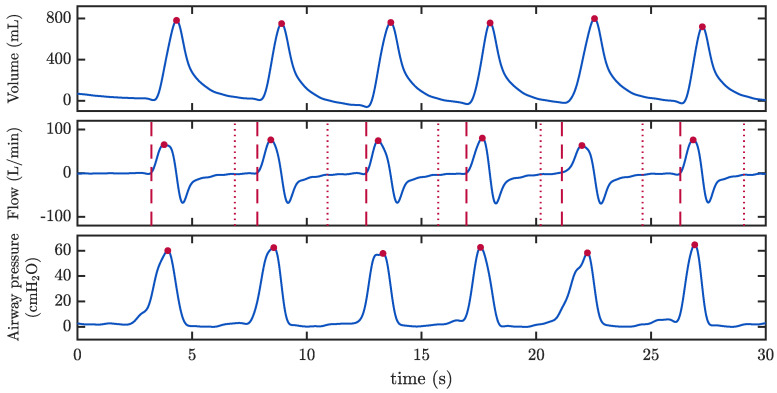
Example of data annotation of additional ventilation parameters in the MUV database. From top to bottom: volume signal with the peak volume per ventilation annotated with red dots; airway flow signal showing the beginning (red dashed lines) and end (red dotted lines) of each ventilation and the peak inspiratory flow (red dots); airway pressure signal with the peak value per ventilation (red dots).

Additional ventilation parameters, not available routinely, were annotated using the airflow, volume and pressure in the MUV database. Signals were low-pass filtered to suppress the artefact induced by CCs. The beginning and end of each ventilation cycle and the peak inspiratory flow were annotated using the airflow. Then, using the volume and pressure signals, the peak volume and peak pressure of each ventilation were annotated. Figure 2 shows an example of the annotated values in a 30 s ventilation interval. Each ventilation was characterized by the following parameters: tidal volume (top panel, red dots), peak inspiratory flow (middle panel, red dots), peak airway pressure (bottom panel, red dots), ventilation duration, and inspiration and expiration time. Minute volume per selected interval was computed. Also, the coefficient of variation in the tidal volume between consecutive ventilations was calculated, that is, the standard deviation of the tidal volume per ventilation expressed as a percent of the mean tidal volume, in blocks of five consecutive ventilations.

#### 2.3.2. Characterization of the Capnogram Oscillation and Its Relationship with Compression Depth

A high incidence of oscillations on waveform capnography during CCs has been previously reported [24,30]. In a previous work, we categorized the oscillation patterns qualitatively as Type I and Type III (located in the expiratory plateau) and Type II (located in the baseline). Then, we conducted an spectral analysis to conclude that the frequency of the oscillations observed in the capnograms matched the frequency of CCs [24]. In the present study, our aim was to objectively measure the amplitude of the oscillations on the capnogram plateau and to assess whether this amplitude was related to the compression depth achieved with each corresponding CC.

For this purpose, the capnogram and the compression depth signal of a convenience sample of TVF&R recordings were examined. Using the annotations of individual ventilations, segments between consecutive ventilations with presence of CCs were identified. The ETCO_2_ value was annotated for each ventilation. Oscillations in the capnogram plateau were isolated and characterized by their amplitude, ${\mathrm{CO}}_{2}{|}_{i}$. Then, the percentage drop of CO_2_ concentration caused by each CCs, was computed as:

(1) $$\Delta {\mathrm{CO}}_{2}{|}_{i}=100\cdot \frac{{\mathrm{ETCO}}_{2}-{\mathrm{CO}}_{2}{|}_{i}}{{\mathrm{ETCO}}_{2}}.$$

**Figure 3:**
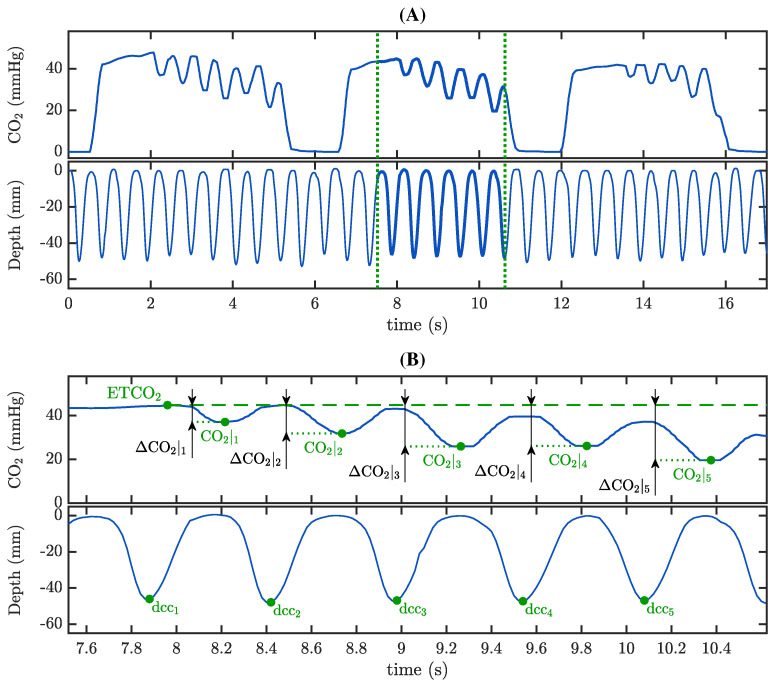
Annotation of parameter $\Delta {\mathrm{CO}}_{2}{|}_{i}$ and corresponding CC-depth, dcc_i_, in the TVF&R database. (**A**) Capnogram tracing (top) showing oscillations caused by CCs, which can be distinguished in the compression depth signal (bottom). (**B**) Expansion of the segment between green dotted lines in panel A, illustrating in detail the annotations.

Each calculated $\Delta {\mathrm{CO}}_{2}{|}_{i}$ was associated with the compression depth of each CC measured in the compression depth signal. Figure 3 shows an example of the annotation procedure. The presence of CCs can be tracked in the compression depth signal, concurrent with the oscillations appearing in the capnogram plateau (Figure 3, panel A). The interval delineated by the green dotted vertical lines is expanded in panel B. Along with the values of $\Delta {\mathrm{CO}}_{2}{|}_{i}$, the maximum CC-depths were annotated.

#### 2.3.3. Characterization of the Capnogram Oscillation and Its Relationship with Associated Pseudo-Volume

**Figure 4:**
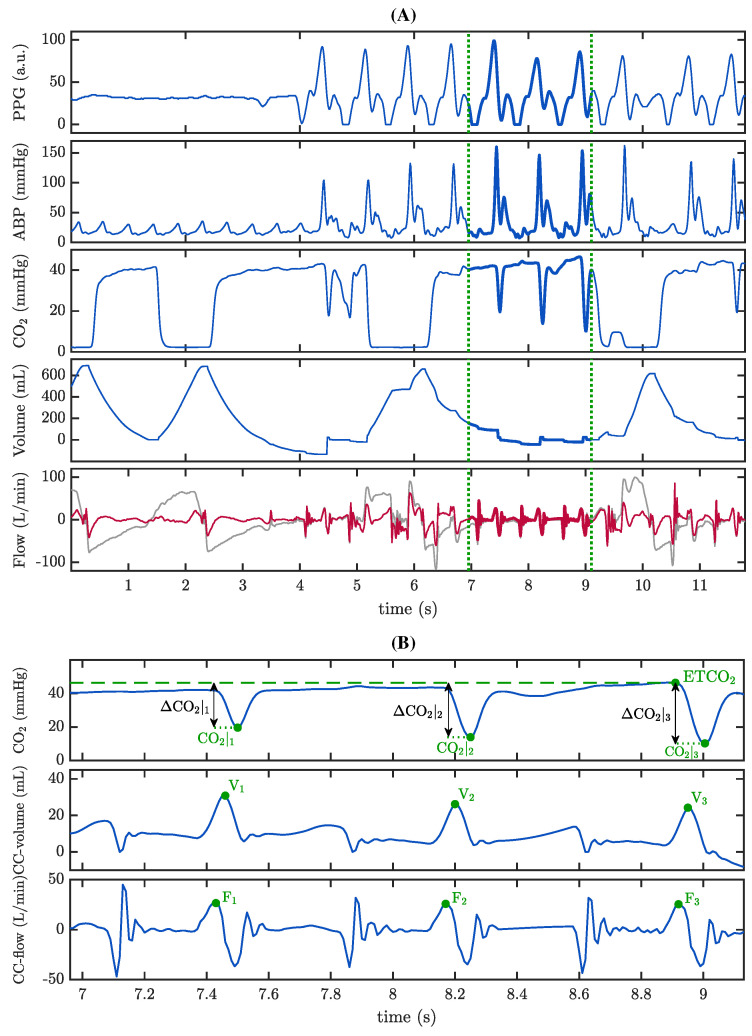
Annotation of parameter $\Delta {\mathrm{CO}}_{2}{|}_{i}$, CC-volume Vi and CC-flow Fi in the MUV database. (**A**) From top to bottom: PPG and ABP signals showing the onset of CCs from second 4 onwards. Third panel: the capnogram tracing is affected by CCs. Forth and fifth panels: volume and airflow signals. The raw flow (grey line) was high pass filtered to enhance CCs activity (CC-flow. red line). a.u.: arbitrary units. (**B**) Expansion of the segment between green dotted lines in panel A, illustrating in detail the annotations.

A second analysis was conducted using the ventilatory signals contained in the MUV database. Using a similar procedure, $\Delta {\mathrm{CO}}_{2}{|}_{i}$ values were calculated. In this case, we wanted to assess the relationship between $\Delta {\mathrm{CO}}_{2}{|}_{i}$ and the tidal volumes caused by CCs. For that purpose, the airflow signal was high-pass filtered to isolate the changes in the airflow caused by CCs (CC-flow component) and the volume attributable to CCs was calculated by integration of the CC-flow signal (CC-volume component). Figure 4A shows an example of the annotation of the segments of interest. The presence of CCs can be tracked in the ABP and PPG signals, concurrent with the oscillations appearing in the capnogram plateau. The grey trace in the bottom panel correspond to the raw airway flow. The red trace depicts the high-pass filtered flow highlighting the changes in the airway flow pattern caused by CCs in isolation. The specific interval delineated by the green dotted vertical lines is expanded in Figure 4B. Along with the values of $\Delta {\mathrm{CO}}_{2}{|}_{i}$, the maximum volume caused by each CC, i.e., passive volume, Vi, and the maximum inspiratory flow value caused by each CC, Fi, were annotated.

**Figure 5:**
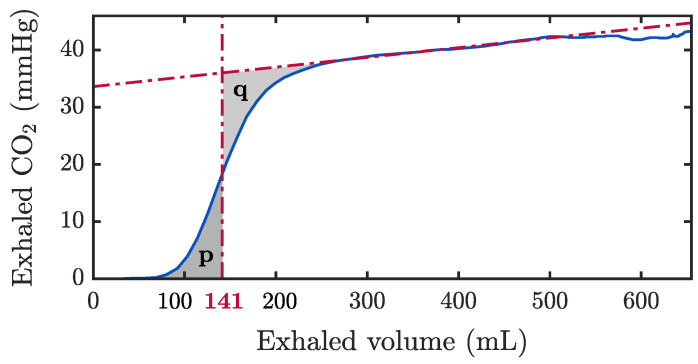
Volumetric capnogram of a ventilatory cycle annotated in the MUV database, representing the exhaled ${\mathrm{CO}}_{2}$ as a function of the exhaled volume in a single ventilation. The red dashed-dotted vertical line, where the areas *p* and *q* are equal, indicates the volume of the anatomical dead space.

In addition, we estimated the anatomical dead space per patient. For this purpose, the volumetric capnogram was derived through the analysis of the capnogram and volume signal of ventilations during pauses in CCs to ensure the integrity of the signals [31]. Figure 5 represents the volumetric capnogram for a single ventilation, i.e., the ${\mathrm{CO}}_{2}$ concentration as a function of volume during exhalation. The vertical red line was obtained by equating the areas *p* and *q*, and the value in the x-axis corresponds to the volume of the anatomical dead space (141 mL in the example), thus providing an effective measurement of the exchanged gas volume lost in the airway.

#### 2.3.4. The Role of Reversed Airflow

We extended our analysis to examine the effects of CCs on the airflow and volume signals and their impact on the waveform capnography along the whole ventilatory cycle. Particularly, we wanted to analyse to what extent changes in the airway flow affect the integrity of the capnogram waveform. Previous authors described the term reversed flow (RF) as the change in the direction of the gas flow during inspiration [32,33]. In our study, we extended the analysis to the whole ventilation cycle. In the absence of CCs, (Figure 2), the airflow is positive during inspiration, reflecting the forward direction of the gas supply; during expiration, the airflow is negative, reflecting the expired gas, and tends to zero at the end of expiration; finally, the airflow is zero in the interval between consecutive ventilations (resting). During CCs, this analysis becomes challenging. We examined our recordings to carefully locate deviations from the conventional airflow pattern attributable to CCs.

#### 2.3.5. Statistical Analysis

Distributions of the ventilation parameters were reported as median (IQR) and depicted using boxplots. Relationships between parameters were analysed using scatter plots and linear regression characterized by the Pearson correlation coefficient, R. Descriptive results are supported by graphical examples.

## 3. Results

### 3.1. Assessment of Ventilation Quality Parameters

We examined a total of 52,654 ventilations from 232 patients from the TVF&R database, with a mean (STD) number of ventilations per patient of 227 (118). Patients were 64 (56–75) years of age, and 29% were females. The first recorded rhythm was shockable (ventricular fibrillation/pulseless ventricular tachycardia) in 38% of cases, pulseless electrical activity in 27%, and asystole in 31%. ROSC was achieved in the field in 59% of the patients and 14% were discharged alive. Median (IQR) ventilation rate was 13.3 (8.6–19.6) vpm. A total of 66.7% of the ventilations were delivered over 10 vpm, 42.4% over 15 vpm, and 23.5% over 20 vpm.

We examined the recordings from the 19 patients included in the MUV database [29]. Only four patients met the requirement of being characterised by signals of sufficient quality for the required annotations during ongoing CCs. A total of 1414 manual ventilations were annotated and characterized. All patients were male and 75% achieved ROSC during the intervention. No further patient data were available.

**Figure 6:**
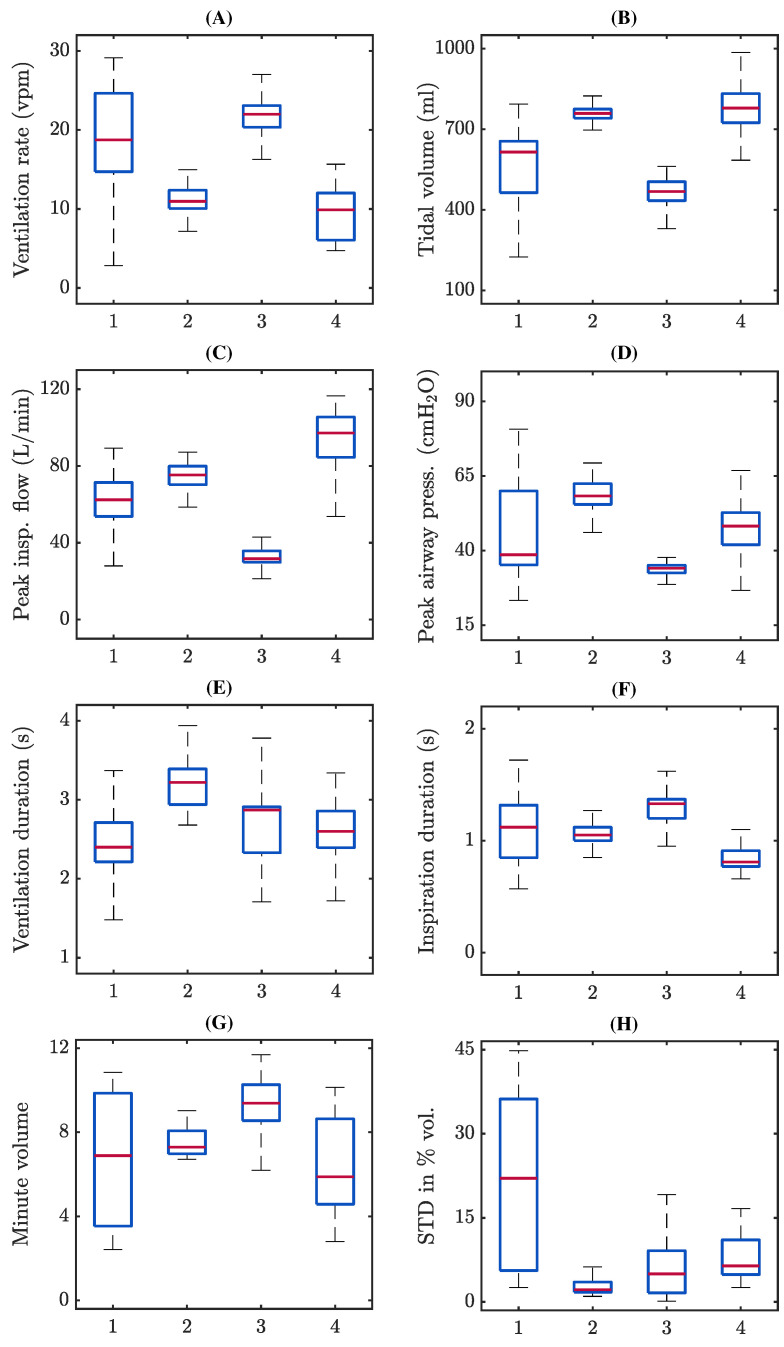
Distributions of the analysed ventilation parameters in the MUV database using boxplots. On each box, the red mark indicates the median, and the bottom and top edges of the box indicate the 25th and 75th percentiles, respectively. The whiskers extend to the most extreme data points not considered outliers. From panel (**A**–**H**): ventilation rate, tidal volume, peak inspiratory flow, peak airway pressure, ventilation duration, inspiration duration, minute volume and coefficient of variation of tidal volume. A large variability was observed intra-patient and inter-patent, confirming that homogeneity in the application of manual ventilation is far from being achieved.

**Figure 7:**
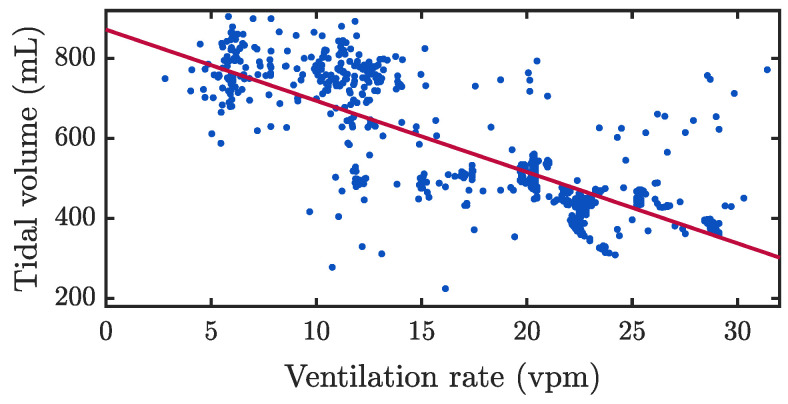
Scatter plot of tidal volume versus ventilation rate (MUV database), with the resulting regression line yielding a Pearson correlation coefficient R of −0.8 (strong negative correlation).

Figure 6 shows the distributions of the annotated ventilation parameters for each case. From panel A to F: median ventilation rate ranged from 9.9 to 22 vpm; median tidal volume from 568 to 778 mL; median peak inspiratory flow between 31.6 and 97.1 L/min; median peak airway pressure between 34.1 and 58.3 cmH_2_O; median ventilation duration from 2.4 to 3.2 s, and median inspiration time from 0.8 to 1.3 s. Panels G and H: median value for minute volume ranged from 5.9 to 9.4 L/min; median coefficient of variation in the tidal volume between consecutive ventilations per episode ranged from 2.2 to 22%. Figure 7 depicts a scatter plot of tidal volume as a function of ventilation rate for all analysed ventilations with the resulting linear regression model, yielding a correlation coefficient R of −0.8.

### 3.2. Capnogram Oscillation Amplitude and Compression Depth

Annotations from a subset of 32 patients from the TVF&R were included in this analysis (19% female, median age 64 (55–75), 38% ROSC, 22% shockable rhythm, 100% death in field). Ventilation rate per episode was 12.3 (8.3–16.7) vpm, compression depth 44.1 (36.0–52.0) mm and compression rate 111.94 (103.45–120.00) cpm. ${\mathrm{ETCO}}_{2}$ was 18.08 (11.15–28.26) mmHg. Percentage of distorted ventilations per case was 42.9% (22.0–67.1%). Globally, $\Delta {\mathrm{CO}}_{2}{|}_{i}$ was 73.7% (43.1–91.9%).

**Figure 8:**
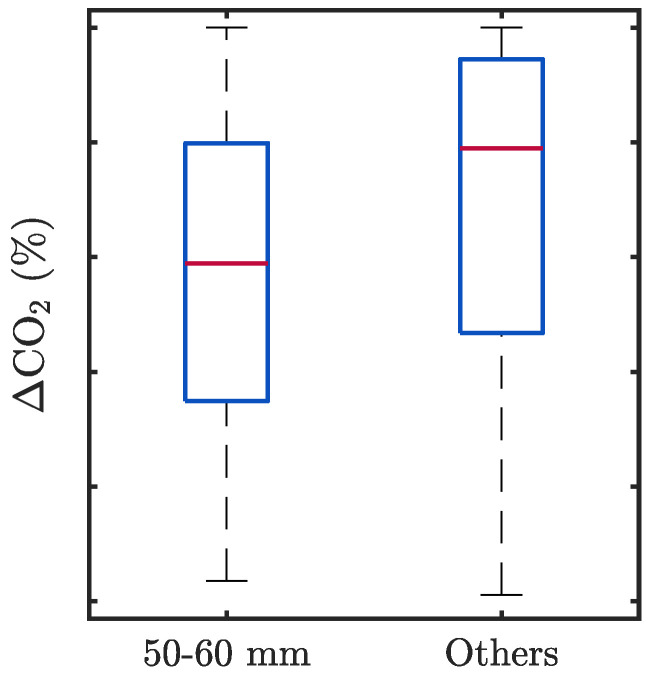
Distributions using boxplots of $\Delta {\mathrm{CO}}_{2}{|}_{i}$ values for CCs with and without adherence to guidelines recommendations for compression depth (from TVF&R database). Despite the absence of correlation between $\Delta {\mathrm{CO}}_{2}{|}_{i}$ and dcc_i_, differences in the two groups were observed.

The correlation between $\Delta {\mathrm{CO}}_{2}{|}_{i}$ and its corresponding compression depth yielded a correlation coefficient R of −0.24 (weak negative correlation). Figure 8 represents the distributions of $\Delta {\mathrm{CO}}_{2}{|}_{i}$ values corresponding to CCs within the range of recommended depth (50–60 mm) and for those outside recommendations.

### 3.3. Capnogram Oscillation Amplitude and Pseudo-Volumes

For this analysis, six patients from the MUV database contained concurrent airflow, ventilation volume, capnogram, and ABP and PPG signals with sufficient quality for the required annotations. Manual CCs were administered in three cases, whereas mechanical CCs were delivered in the other three. All patients were male, and four of them achieved ROSC at some point during the intervention (no further patient data were available). In the episodes with mechanical CCs, no passive ventilations generated by CCs were observed, i.e., there was neither CC-artefact in the capnogram nor CC-volume component in the volume signal. Durations of the episodes with manual CCs were 55, 60 and 83 min, with median ventilation rates of 18.8 (14.7–24.6), 22.0 (20.3–23.1) and 9.9 (6.1–12.0) vpm, respectively. In these episodes, we annotated 190 segments containing passive ventilations caused by CCs (24, 37 and 129) with a total of 1490 CCs (96, 299 and 1095), respectively.

**Figure 9:**
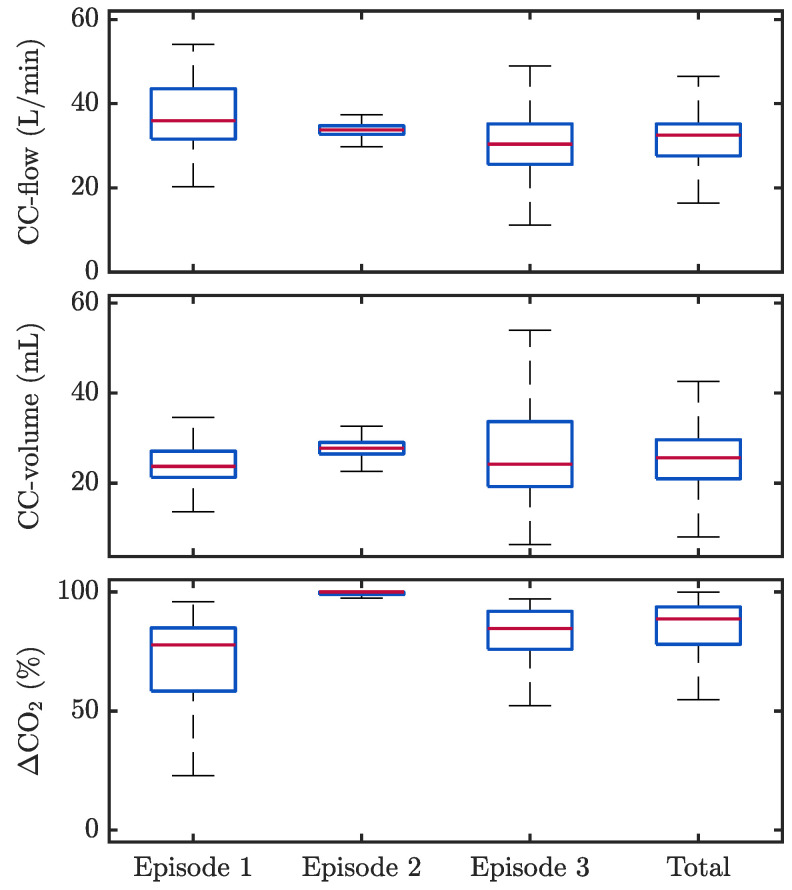
Boxplots of the maximum CC-flow (**top**), CC-volume (**middle**), and $\Delta {\mathrm{CO}}_{2}{|}_{i}$ values (**bottom**) per patient and for the whole population (MUV database). A noticeable variability between patients was observed. Note the stable measurements in patient 2, in contrast with the dispersion in the other cases.

Figure 9 shows the distributions for CC-flow (Fi), CC-volume (Vi) and $\Delta {\mathrm{CO}}_{2}{|}_{i}$ per episode and for the whole population. Globally, median $F_{i}$ was 32.5 (27.6–35.1) L/min, median $V_{i}$ was 25.6 (21.0–30.0) mL, and median $\Delta {\mathrm{CO}}_{2}$ was 88.7 (78.0–93.7)%.

According to the procedure depicted in Figure 5, median anatomic dead space volumes per patient were 149.2, 138.8 and 151.7 mL, respectively.

**Figure 10:**
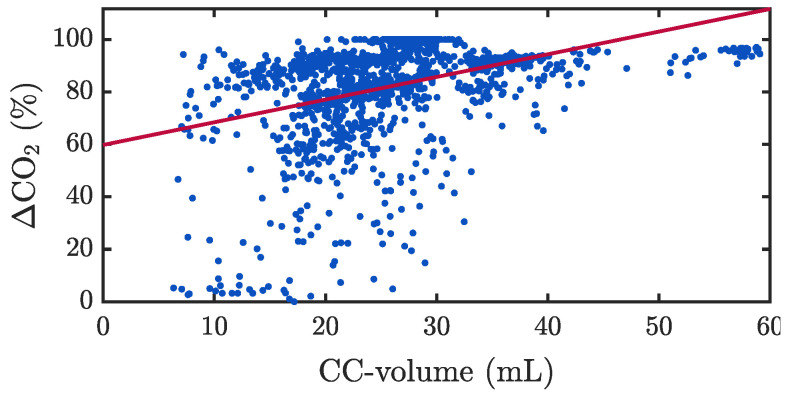
Scatter plot of $\Delta {\mathrm{CO}}_{2}{|}_{i}$ versus passive volume, Vi, caused by CCs (MUV database). The red line corresponds to the linear regression model, which yielded a Pearson correlation coefficient R of 0.4 (moderate positive correlation).

**Figure 11:**
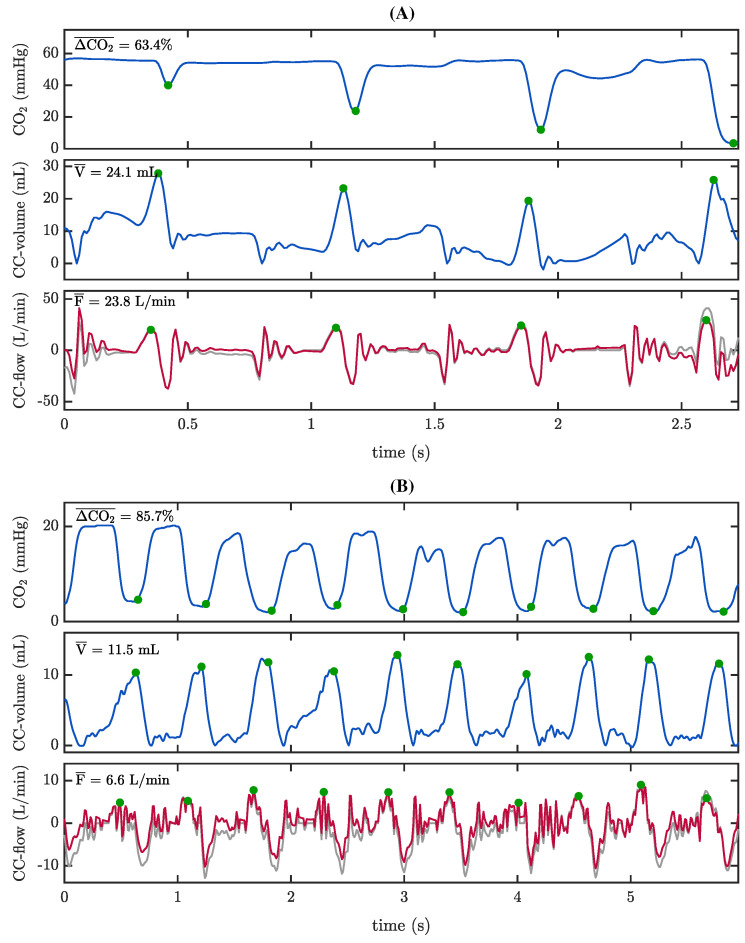
Examples of distorted capnogram plateau (similar to the representation in Figure 4B), showing different behaviour in relation to ${\mathrm{CO}}_{2}$ drop, passive CC-volume and CC-flow (green dots) (MUV database). (**A**) Increasing $\Delta {\mathrm{CO}}_{2}$ values despite stable CC-volumes. (**B**) Stable $\Delta {\mathrm{CO}}_{2}$ values and CC-volumes. Top, left labels: Mean values of the annotations along the whole time interval.

Figure 10 presents a scatter plot of $\Delta {\mathrm{CO}}_{2}{|}_{i}$ as a function of $V_{i}$ and the obtained linear regression model, with a correlation coefficient R of 0.4. Figure 11 shows two segments with differences in the relationship between $V_{i}$ and $\Delta {\mathrm{CO}}_{2}{|}_{i}$. The first segment (Figure 11A) showed similar passive volumes in the four applied CCs, 24.1 mL on average. However, $\Delta {\mathrm{CO}}_{2}$ caused by the first compression was 43.2%, while it increased to 62.4%, 72.5% and 75% in the following three CCs, respectively. The second segment (Figure 11B) presented ten CCs with an average passive volume of 11.5 mL generating an average $\Delta {\mathrm{CO}}_{2}$ of 85.7%, with no observable differences between consecutive CCs.

### 3.4. The Role of Reversed Airflow

**Figure 12:**
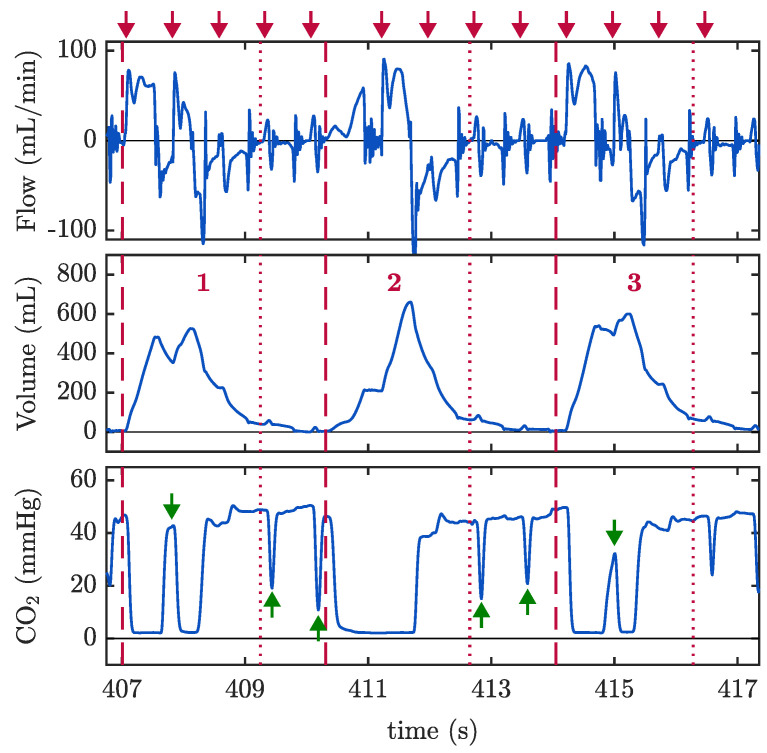
RF events causing capnogram oscillations (bottom panel, green arrows). Red arrows on top of the figure indicate individual CCs. Red dashed lines indicate the onset of inspiration and red dotted lines the offset of expiration. Events of RF cause distortion in the volume signal (middle panel) that affect differently the capnogram pattern. Upward oscillations in the capnogram baseline (green down-arrowhead) and downward oscillations in the plateau (green up-arrowhead) resemble pseudo-expirations and pseudo-inspirations, respectively. Segments extracted from the MUV database.

For this exploratory analysis, we reviewed the capnogram, volume and airflow signals during CCs of all the annotated ventilations in the MUV database for assessing occurrences of RF along the whole ventilation cycle (inspiration, expiration, and rest). During CCs, spiky patterns were observed in the airflow at the instants when the chest is compressed and released, disturbing the normal airflow pattern. Undesired changes in the airflow polarity caused alterations in the volume and in the capnogram (see Figure 12). When the airflow was negligible (resting interval between consecutive ventilations), spikes on airflow originated by CCs generated small volumes (passive ventilations) which were responsible for the characteristic oscillations in the capnogram plateau (described as Type I and III in our previous work [24]). This was observed in 70% of the examined ventilations. During positive airflow (inspiration) and negative airflow (expiration), events of RF caused opposed movement of gases in the 80% of the examined ventilations. However, only when RF caused noticeable volume drops during inspiration did this result in significant rises CO_2_ concentration (Type II oscillations in our study [24]). During the onset of expiration phase prior to the capnogram plateau, RF events did not have a significant impact on either volume or capnogram, although little distortions in the waveform were observed.

## 4. Discussion

High-quality CPR plays a key role in survival from cardiac arrest. Over the years, resuscitation guidelines have emphasized on the quality of CCs, defining clear metrics of adequate CC performance with well-established targeted goals for CC depth and rate. Widespread deployment of systems for real-time monitoring of the quality of CCs in the field have made it possible to measure, review and improve quality of care. The guidelines also reinforce the importance of avoiding excessive ventilation rates, but recognise the limitations of the capnography signal and the lack of technology to measure other important parameters such as tidal volumes or inspiratory pressures. Due to this lack of robust evidence, the optimization of ventilation during CPR remains a challenge.

### 4.1. Assessment of Ventilation Quality Parameters

Currently, the ventilation parameter most commonly monitored during resuscitation is ventilation rate, usually derived from real-time processing of waveform capnography. Studies reveal a tendency to over-ventilate the patient, that is, to apply excessive ventilation rates [3]. Technical limitations complicate monitoring other relevant ventilation parameters such as tidal volumes, airway flow and pressure, or inspiratory/expiratory durations. Studies reporting these parameters in real cardiac arrest interventions are scarce and, in general, involve a reduced number of patients [34,35].

Our study confirmed the tendency to apply ventilation rates higher than the recommendation of 10 breaths/min after intubation of OHCA patients. We observed a significant dispersion among the measured values, supporting the need to further investigate on the reasons for this lack of homogeneity. When we replicated the analysis for the MUV patients during the ICU-CPR, we also observed large inter-patient deviations, with patients being ventilated at large ranges of ventilation rates, some coming close or even exceeding 20 vpm. The same patient could also be ventilated with a very high range of ventilation rate. The use of a metronome to conveniently guide the frequency of ventilations could help improve adherence to recommendations. In our opinion, further efforts should be made to develop feedback systems on the quality of the ventilation rate. Either the capnogram or the transthoracic impedance can be used for this purpose, although both signals have to be pre-processed to remove the fluctuations that CCs cause in the waveforms to emphasise the ventilation component [36,37].

Regarding the relationship between ventilation rate and volume, we generally found an inversely proportional relationship between tidal volumes and ventilation rates (Figure 7). However, some patients who were routinely ventilated at 10 vpm also presented volumes well above the recommended ones. In contrast, some patients hyperventilated at rates close to 20 vpm maintained the recommended volumes. We also observed a positive association between tidal volumes and peak inspiratory flow, but a weaker association between peak flow and peak inspiratory pressure (Figure 6). The timing of ventilation was more consistent. Median ventilation duration ranged from 2.4 to 3.2 s, while inspiratory time varied between 0.8 and 1.3 s, quite close to the 1 s guideline. Volume changes between adjacent ventilations were in general below 10% of the mean volume, although we detected one patient with a variation of 22%.

Despite the limitations of the data, the variability in ventilation rate had a correspondence with the variability of other key parameters. Our findings suggest that there is a need to deploy systems that can measure more information than only ventilation rate. A recent pilot study by Yang et al. [21] showed that measuring respiratory mechanics in the pre-hospital setting was feasible. Recently described feedback devices could assist rescuers during ventilation, although they were tested in simulated manikin environments [38,39]. Nonetheless, there is still a long way to go before reliable, useful and cost-effective devices are clinically available [3].

### 4.2. What We Know about Oscillations in the Capnogram Plateau during Chest Compressions

Distortion of the capnogram during CCs impedes accurate detection of ventilation occurrences, therefore affecting the accuracy of the measurement of ventilation rate. In a previous study, we demonstrated the accurate synchronization of the oscillations in the capnogram plateau and the chest compression rate [24]. In the present study, we found a weak correlation between the amplitude of the oscillation and the depth of the chest compression. Therefore, we focused on other ventilatory signals that are not routinely recorded at present to look for possible relationships.

Several studies have demonstrated and quantified the generation of passive ventilations during CCs, caused by changes in intrathoracic pressures and consequent movement of gas in the airway [5,22,27,40,41]. In all the analysed manual CCs, there was full concurrence among the compression–decompression manoeuvre, the appearance of a small added volume during expiration and an oscillation in the capnogram expiratory plateau. Previous studies on OHCA patients receiving compression-only CPR showed median tidal volumes varying from 7.5 mL [28] to 41.5 mL [27]. Our results were in line with those reported in pre-hospital settings [22], with a median passive volume of 25.6 mL, and low dispersion among patients. These small volumes are clearly insufficient for adequate patient ventilation. This fact was confirmed by the large differences between measured passive volumes and the patient anatomical dead spaces (139–152 mL). Globally, CC flow values were relevant compared to the raw flow (Figure 9) but generated low CC volumes due to the short duration of the CC flow changes (Figure 4). Furthermore, the CC-flow waveform has two different phases in each individual CC, one varying faster than the other (compression vs. decompression or release phase). Only the slower burst appearing during chest decompression generated gas movement into the airway, sufficient to decrease ${\mathrm{CO}}_{2}$ concentration and distort the capnogram (Figure 11). When CC-flow was less impulsive, the waveform presented only a single identifiable phase in each CC. These differences may be attributable to the influence of both the anatomical characteristics of each patient and the rescuer’s CPR delivery.

In spite of these small induced flows and volumes, the oscillations caused by CCs in the capnograms were of high amplitude, with a global median value of 73.7% in the TVF&R database and of 88.7% in the MUV database. This confirmed their negative impact on adequate clinical interpretation of the capnogram in terms of ventilation rate feedback and accurate measurement of ETCO_2_ [24,25,42,43]. There was a slight tendency of increasing oscillation with increasing volume (Figure 10), but no direct relationship was observed locally, as reflected in the examples of Figure 11. Such behaviour suggests a certain randomness in the amplitude of these oscillations. Recent studies have investigated this phenomenon using bench models and human cadaver models [41,44] and have related it to the physiological concept of lung volume reduction and its associated thoracic airway closure. They hypothesized that the appearance or absence of oscillations on the capnogram may be indicative that the patient’s airway is open or closed, respectively. In our study, we found that the amplitude of the CO_2_ oscillation was higher (airway open) when CCs were provided with a depth outside the recommended range. This contradictory finding merits further study.

Ultimately, capnogram oscillations during CCs can be seen as either an interference that needs to be removed to enhance the clinical interpretation of the capnogram or a desired phenomenon to observe, indicative of airway opening. Oscillations can be removed using linear filtering to enhance ventilation detection [36] and the envelop of the capnogram can be restored to allow accurate ETCO_2_ measurement [45]. Measuring the amplitude of oscillations in the capnogram is feasible. Efforts should be made to transfer these techniques to new real-time guidance systems.

### 4.3. The Role of Reversed Airflow during Chest Compressions

Finally, asynchronous ventilations during ongoing CCs produced a high incidence of reversed flow. Between ventilations, when the airflow is close to zero, CCs induced airflow spikes and pseudo-volumes (passive ventilation), eventually causing oscillations in the capnogram plateau, as we have already described before. More importantly, during positive and negative airflow (corresponding to inspiration and expiration, respectively), events of RF caused opposed movement of gases in the majority of the examined ventilations. However, we observed that only when RF caused noticeable volume drops during inspiration did this result in significant rises in CO_2_ concentration (Type II oscillations). During the expiration phase, RF events did not have a significant impact on volume or capnogram, although a small distortion was observed. Our findings are in line with the observations by Duchatelet et al. [32] reporting a high incidence of reversed flow in most of the OHCA patients included in the study, and recently by Van Den Daele et al. [33] reporting airflow fragmentation in all analysed patients. In the present study, we have taken a small step forward by characterising these occurrences in the different phases of the ventilation cycle, relating them to changes in volume and their implication on the capnogram. Despite our limited dataset, we have observed similar results in our study and agree that this fragmentation may affect proper ventilation, leading to exhalation of gases during inspiration and errors in the measurement of delivered tidal volumes. As waveform capnography is the only current gold standard for monitoring ventilation in out-of-hospital settings, distortions in its waveform due to these phenomena should be studied further.

### 4.4. Limitations

The major limitation of the study is the relatively low number of patients for whom ventilation parameters have been assessed and compared. A second limitation is its retrospective nature, due to which full control on the signal recordings was not possible. However, the observed variability in ventilation rate in our patient population might anticipate a similar variability in other components during manual ventilation. Finally, the data were acquired some time ago. This is in part because such databases are hard to obtain, and also since the process of data gathering, curation, review and annotation was long and costly. Updating these results with recent data would, nevertheless, improve reliability and applicability of the study.

## 5. Conclusions

Our study confirmed lack of adherence between routinely measured ventilation rates and the guidelines recommendation in intubated patients during CPR, as well as substantial dispersion in additional and relevant ventilation parameters, which are not often recorded in the field nowadays due to lack of adequate devices. The development of ventilation feedback systems, similar to those currently available for chest compression quality monitoring, would be of great benefit. Oscillations caused by CCs on capnograms were frequent, but their amplitudes did not correlate with compression depth or with associated tidal volumes. CCs caused changes in the airflow direction during inspiration, expiration and in the interval between ventilations, sufficient to generate volume changes and causing oscillations on capnograms. Further research is justified to assess the impact of these findings on ventilation quality during CPR.

## Data Availability

Data are available upon request to the corresponding author.

## Abbreviations

The following abbreviations are used in this manuscript:

CPR	Cardiopulmonary resuscitation
CCs	Chest compressions
ROSC	Restoration of spontaneous circulation
ALS	Advanced life support
ETCO_2_	End-tidal CO_2_
OHCA	Out-of-hospital cardiac arrest
TVF&R	Tualatin Valley Fire & Rescue
EMS	Emergency medical services
ROC	Resuscitation outcomes consortium
OHSU	Oregon Health & Science University
MUV	Medical University of Vienna
TI	Transthoracic impedance
PPG	Photoplethysmography
ABP	Arterial blood pressure
AOI	Airway opening index
RF	Reversed airflow
